# Effects of sample age and storage temperature on the flow cytometric diagnosis of chronic lymphocytic leukaemia in South Africa

**DOI:** 10.4102/ajlm.v14i1.2688

**Published:** 2025-05-31

**Authors:** Shaun M. Grobler, Anne-Cecilia van Marle

**Affiliations:** 1 Department of Haematology and Cell Biology, Faculty of Health Sciences, University of the Free State, Bloemfontein, South Africa; 2 Department of Haematology, National Health Laboratory Service, Bloemfontein, South Africa

**Keywords:** chronic lymphocytic leukaemia, atypical chronic lymphocytic leukaemia, flow cytometry, Matutes score, pre-analytical variables, storage time, storage temperature

## Abstract

**Background:**

Chronic lymphocytic leukaemia (CLL) is a haematological neoplasm with characteristic flow cytometric immunophenotyping. Pre-analytical variables impact the quality and reproducibility of flow cytometric data, which could alter the diagnosis from CLL to atypical CLL (aCLL).

**Objective:**

This study investigated the effects of pre-analytical variables, specifically sample age and storage temperature, on the stability of key antigens used in the diagnosis of CLL.

**Methods:**

Serial flow cytometric analyses were performed from January 2022 to March 2023 on blood samples of 10 CLL patients from the Universitas Academic Hospital Haematology Clinic in Bloemfontein, South Africa. Samples were stored at room and refrigerator temperatures and analysed at baseline, 24 h, 48 h, 72 h and 96 h. We recorded the percentage and intensity of antigen expression of CLL makers, including CD5, CD20, CD23, CD79b, CD200 and sIgM, and assessed whether these affected the adapted and modified Matutes scores.

**Results:**

Statistically significant changes were observed in CD5 (*p* = 0.028), CD23 (*p* = 0.003) and CD200 (*p* = 0.005) expression, with better stability at refrigerator temperature. Two samples showed changes in both Matutes scores by 24 h, irrespective of storage temperature. By 48 h, scores changed to aCLL in six room-temperature and four refrigerated samples. A majority shift in diagnosis to aCLL (modified Matutes: *n* = 8/10; adapted Matutes: *n* = 7/10) was observed at 96 h for refrigerated samples.

**Conclusion:**

These findings indicate that pre-analytical variables influence antigen stability in CLL samples, with better preservation at refrigerator temperature, recommending analysis within 48 h of collection.

**What this study adds:**

This study highlights the impact of pre-analytical variables on the flow cytometric diagnosis of CLL. Extended room temperature storage alters antigen expression, shifting Matutes scores and potentially affecting the final diagnosis. The findings emphasise optimised sample handling, for improved diagnostic accuracy in laboratory medicine.

## Introduction

Chronic lymphocytic leukaemia (CLL) is a clonal haematological disorder consisting of immunologically inept, small, monomorphous and well-differentiated B-lymphocytes that aberrantly express the antigens CD5 and CD23 on their surfaces.^1^ A sustained (≥ 3 months) monoclonal B-lymphocytosis of ≥ 5 × 10^9^/L in the peripheral blood, with characteristic morphological and immunophenotypic features, is required to establish the diagnosis of CLL.^2^

Immunophenotyping not only confirms the diagnosis of a mature, clonal B-cell neoplasm but also helps to differentiate CLL from other B-cell lymphoproliferative disorders (B-LPDs). Notwithstanding substantial advances in cytogenetics and molecular biology, morphology and immunophenotyping remain the benchmark for diagnosing mature B-LPDs.^3^

The Matutes score, developed in 1994, uses five surface markers (CD23, CD5, FMC7, surface immunoglobulin [sIg], and CD22) to diagnose CLL, with scores of 4 or 5 indicating CLL, and scores below 4 suggesting non-CLL; a score of 3 implies atypical CLL (aCLL).^4,5,6^ In 1997, a modification known as the Moreau score replaced CD22 with CD79b to better distinguish CLL from other B-LPDs.^4,5,6^ Cooperative groups, including the EuroFlow consortium, now omit FMC7 and use CD20 to streamline testing without compromising diagnostic accuracy.^5,7,8^ CD200, identified in 2009, further improved the score’s accuracy, distinguishing CLL from mantle cell lymphoma.^9^ An adapted Matutes score that includes CD200 has been shown to achieve nearly 100% diagnostic accuracy for CLL, underscoring the value of these refinements.^10^ The concept of aCLL, initially described by Criel et al.,^11^ refers to a B-LPD with atypical morphology, including larger CLL cells with pleomorphic nuclear contours. The revised 4th edition of the World Health Organization Classification of Tumours of Hematopoietic and Lymphoid Tissues describes aCLL as having at least 10% circulating ‘CLL cells’ with prominent nuclear clefts and folds.^12^

The incidence of aCLL is reported to be 7% among all B-LPDs diagnosed by flow cytometry.^13^ Atypical CLL is described as having a lower Matutes score owing to the unusually brighter expression of sIg or CD79b, lack of CD5 or CD23, or positivity for FMC7/CD20.^11,13,14^ These immunophenotypic changes may, however, be secondary to trisomy 12, acting as a confounding variable.^11,14^

Whether the distinction between CLL and aCLL is clinically significant remains controversial, although aCLL has been associated with a more aggressive clinical course and a higher probability of disease progression.^14^ Owing to the lack of a concrete standardised concept of aCLL,^15^ and ambiguous definitions in the successive World Health Organization classifications,^3,16^ aCLL is now used more generically to denote forms with an unusual immunophenotype, regardless of blood smear findings.^16^

The impact of pre-analytical variables on the quality and reproducibility of flow cytometry data is well described.^17,18,19,20^ Parameters such as the type of anticoagulant in which samples are collected, the storage time and the storage temperature of samples before analysis, were found to affect absolute cell counts and antigen expression. However, these effects have primarily been assessed on healthy subjects’ blood samples. Extensive comparative studies to determine the optimal pre-analytical flow conditions for B-LPD samples are lacking.^17,19,20^

Universitas Academic Hospital National Health Laboratory Service (NHLS) is in the city of Bloemfontein in the Free State province of central South Africa. Universitas Academic Hospital NHLS serves as the referral laboratory for the Free State, Northern Cape and North West provinces, and provides diagnostic and laboratory services for a population of 8.41 million people, occupying a total geographic surface area of 607 596 km^2^.^21^ Universitas Academic Hospital NHLS often receives blood samples requiring immunophenotyping from distant peripheral clinics, which only reach the laboratory 3–5 days after collection.

Consequently, Universitas Academic Hospital NHLS frequently diagnoses immunophenotypically ‘atypical CLL’ cases, where the morphological assessment of these blood samples supports ‘typical’ CLL. This raises the question of whether we truly see more atypical cases or whether the sample age and storage conditions significantly affect the percentage antigen expression and intensity of expression on lymphoid cells in haematological malignancies.

The aim of our study was to measure objectively the effects of sample age (from time of collection to time of analysis) and storage conditions (room versus refrigerator temperature) on the percentage antigen expression and the intensity of expression of surface antigens in CLL. We wanted to determine the optimal storage conditions to produce quality results and establish a cut-off time after which samples are considered unsuitable for reliable diagnoses.

## Methods

### Ethical considerations

The study was conducted in accordance with the Declaration of Helsinki and approved by the Health Sciences Research Ethics Committee of the University of the Free State (reference number UFS-HSD2021/1257/3011), Free State Department of Health and the National Health Laboratory Service. As per the institutional review board, no informed consent was required as there was no interaction between the principal investigator and the patients, nor did the study’s outcome affect patients’ well-being or medical care. The blood samples used in this study were the surplus blood of routine samples collected by trained nursing staff during the patient’s scheduled haematology clinic visits, independent of our research. The researchers did not have access to patient sociodemographic or clinical details.

### Study design and patient selection

Whole blood samples in vacutainers containing ethylenediamine tetra-acetic acid (EDTA) (Becton Dickinson [BD], San Jose, California, United States) were obtained by designated clinic personnel from patients with confirmed diagnoses of CLL attending the Universitas Academic Hospital Haematology Clinic, Bloemfontein, South Africa, between January 2022 and March 2023. The blood samples used in this study were the surplus blood of the routine blood samples collected by trained nursing personnel during the patients’ haematology clinic visit, independent of our research.

Full blood count and peripheral blood smear results were screened to identify eligible samples. These included samples with morphologically detectable disease, regardless of whether the patients were newly diagnosed or receiving treatment. The EDTA samples were immediately de-identified and assigned study numbers to ensure patient confidentiality, after which baseline (within 12 h of collection) flow cytometric analysis was performed. Only samples with a baseline immunophenotype of typical CLL were included in the study.

**FIGURE 1:**
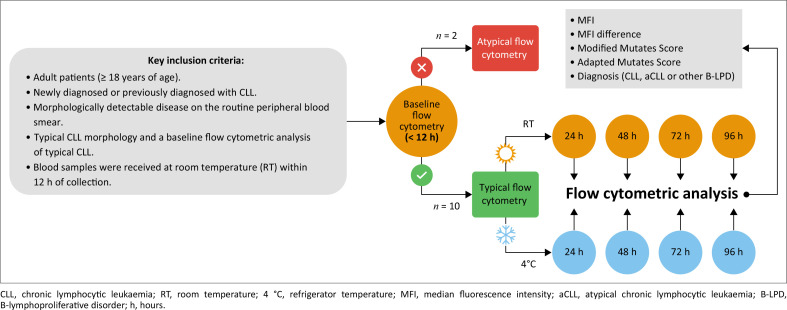
Schematic representation of the study design, Universitas Academic Hospital, Bloemfontein, South Africa, January 2022 to March 2023.

Following baseline flow cytometry, each sample was then divided into two separate specimen tubes: one stored at refrigerator temperature (4 °C) and the other at room temperature (RT), defined as 23 °C in NHLS laboratories.^22,23^ These two aliquots per sample were then analysed after 24 h, 48 h, 72 h and 96 h of storage at the respective temperatures. All flow cytometric analyses were performed on the FACSCanto™ II (BD, San Jose, California, United States). The percentage antigen expression, median fluorescence intensity (MFI) and difference in MFI for each marker were recorded, as shown in Figure 1, which illustrates the study design.

### Sample processing

Sample preparation was done according to the EuroFlow protocols.^22^ Because of the inclusion of surface membrane immunoglobulins (sIgM, sKappa, sLambda), a wash-stain-lyse approach was followed. In brief, 3000 µL of phosphate-buffered saline was mixed with 300 µL of EDTA whole blood, followed by centrifugation at 540 g for 5 min in a Heraeus Megafuge 8 centrifuge (Thermo Scientific Inc., Waltham, Massachusetts, United States).

Thereafter, the supernatant was discarded and these steps repeated once more. The cell pellet was then resuspended in 300 µL of phosphate-buffered saline. The correct volume of cells required per tube was calculated using Equation 1:


Volumeofcellsuspension=1/whitecellcountonfullbloodcounanalyser×1000μL [Eqn 1]

This calculated volume was incubated for 15 min at RT in the dark with 10 µL of each of the following monoclonal antibodies: CD5, CD19, CD20, CD23, CD43, CD45, CD79b, CD200, sIgM, sKappa, and sLambda. The chosen antibody panel (Table 1) was adapted from the EuroFlow Lymphoid Screening Tube and B-LPD panels including those markers considered crucial in the diagnosis of CLL.^1,5,7^

**TABLE 1:** Antibody panel design for flow cytometric analysis of patients attending the Universitas Academic Hospital Haematology Clinic, Bloemfontein, South Africa, January 2022 to March 2023.

Tube	FITC	PE	PerCP Cy5.5	PE Cy7	APC	APC-H7	Pac B	Pac O
1	CD23 (Dako)	-	CD79b (BD Biosciences)	CD19 (Beckman Coulter)	CD200 (eBioscience)	CD43 (BD Biosciences)	CD20 (BioLegend)	CD45 (Invitrogen)
2	sIgM (Dako)	sKappa (Cytognos)	CD5 (BD Biosciences)	CD19 (Beckman Coulter)	-	sLambda (BD Biosciences)	CD20 (BioLegend)	CD45 (Invitrogen)

FITC, fluorescein isothiocyanate; PE, phycoerythrin; PerCP Cy5.5, peridinin-chlorophyll-protein cyanine 5.5; PE Cy7, phycoerthrin cyanine 7; APC, allophycocyanin; ApC-H7, allophycocyanin hilite 7; Pac B, Pacific blue; Pac O, Pacific orange; sIgM, surface immunoglobulin M; sKappa, surface kappa; sLamba, surface lambda; CD, cluster of differentiation.

The cells were then lysed with 2000 µL of diluted BD FACS™ Lysing Solution (BD Biosciences, San Jose, California, United States) and incubated at RT in the dark for another 15 min. Following centrifugation at 540 g for 5 min, the supernatant was discarded, and the cell pellet finally resuspended in 500 µL of phosphate-buffered saline for analysis on the FACSCanto™ II (BD, San Jose, California, United States).

### Sample analysis and interpretation

Analysis was conducted using the BD FACSDiva™ version 8 (BD, San Jose, California, United States) and Infinicyt™ version 2.0 (Cytognos, Santa Marta de Tormes, Salamanca, Spain) software.

**FIGURE 2:**
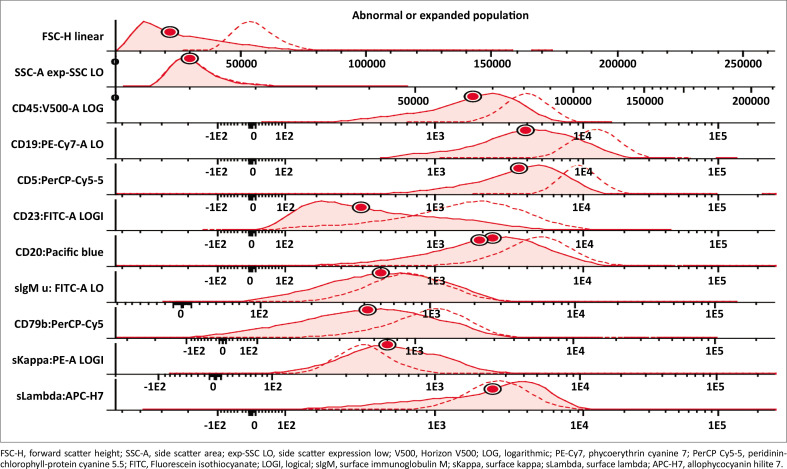
Example of change in median fluorescence intensity of one subject from baseline (dotted histogram) compared to 96 h (solid histogram) at Universitas Academic Hospital, Bloemfontein, South Africa, January 2022 to March 2023.

The lymphocyte gate (forward versus side light scatter), CD45 and CD19 against side scatter were employed for gating. Normal T-lymphocytes were used as internal negative reference populations (NRP), and, where necessary, with markers such as CD5, typically expressed by T-cells, neutrophils were employed as the NRP. Positive designation for markers were assigned if expression was present in > 20% of monotypic B cells^24,25,26^ or when the fluorescent signal for a particular marker was greater than that of the internal NRP. Negative staining was assigned when the fluorescence of any given marker was similar to that of the NRP (qualitative assessment).^22,26^ Median fluorescence intensity, and specifically MFI difference, that is, the intensity of expression in monotypic B cells compared to that of the internal reference populations in arbitrary units provided by the analysis software, and converted to logarithms, was used for quantitative assessment. Table 2 and Figure 2 represent an example of the MFI measurements for the different markers in one CLL sample.

**TABLE 2:** An example of the median fluorescence intensity measurements for the different markers in one sample of a patient with chronic lymphocytic leukaemia at Universitas Academic Hospital, Bloemfontein, South Africa, January 2022 to March 2023.

Marker	MFI of the POI	Log of MFI POI	MFI of the NRP	Log of MFI NRP	Log MFI difference between POI and NRP	Expression
CD45	3529.12	3.55	93.84	1.97	1.58	Moderate
CD19	13 152.35	4.12	41.76	1.62	2.50	Bright
sKappa	366.40	2.56	193.39	2.29	0.28	Negative
sLambda	2381.90	3.38	11.51	1.06	2.32	Bright
sIgM	697.44	2.84	64.96	1.81	1.03	Moderate
CD5	8817.29	3.95	360.00	2.56	1.39	Moderate
CD23	1481.46	3.17	53.11	1.73	1.45	Moderate
CD20	4460.42	3.65	103.80	2.02	1.63	Moderate
CD79b	1142.32	3.06	73.54	1.87	1.19	Moderate
CD43	3954.49	3.60	907.00	2.96	0.64	Dim
CD200	1221.85	3.09	94.04	1.97	1.11	Moderate

Note: MFI difference < 1 logarithm brighter than the NRP = dim expression; MFI difference 1 to < 2 logarithms brighter than the NRP = moderate expression; MFI difference ≥ 2 logarithms brighter than the NRP = bright expression.

MFI, median fluorescence intensity; POI, population of interest; NRP, internal negative reference population; CD, cluster of differentiation; sKappa, surface kappa; sLambda, surface lambda; sIgM, surface immunoglobulin M.

In addition to the positive or negative designation, markers were further described according to the intensity of the positive signal as either dim, moderate, bright or variable by comparing the fluorescent signal, expressed in logarithms, of each marker to the NRP. Dim staining was defined as mildly increased when compared to the NRP (less than one logarithm brighter than the NRP). Moderate staining was at least one logarithm brighter than the NRP, and bright staining was defined as at least two logarithms brighter than the NRP (Table 2).^26^

Two CLL scoring systems, the Moreau score and the adapted Matutes score, were applied to the flow cytometric data for the various intervals and temperatures to determine a change in the diagnosis from CLL (Table 3).^1,4,6,10^ The diagnosis of CLL, employing the Moreau and the adapted Matutes scores, necessitates the observation of dim (1 point) or bright (0 points) expression of certain markers (sIg and CD79b). Consequently, instances of transition to moderate expression did not result in point deduction within the scoring framework.

**TABLE 3:** Modified Matutes/Moreau and adapted Matutes scores for the diagnosis of chronic lymphocytic leukemia at Universitas Academic Hospital, Bloemfontein, South Africa, January 2022 to March 2023.

Marker	Score points†	
	1	0
**Modified Matutes/Moreau score**		
sIg	Dim	Bright
CD5	Positive	Negative
CD23	Positive	Negative
FMC7/CD20	Negative	Positive
CD79b	Dim	Bright
**Adapted Matutes score**		
sIg	Dim	Bright
CD5	Positive	Negative
CD23	Positive	Negative
CD200	Positive	Negative

*Source*: Adapted from Moreau EJ et al.^6^ and Jalal SD.^10^ Please see the reference list of this article, Grobler SM, Van Marle A-C. Effects of sample age and storage temperature on the flow cytometric diagnosis of chronic lymphocytic leukaemia in South Africa. Afr J Lab Med. 2025;14(1), a2688. https://doi.org/10.4102/ajlm.v14i1.2688, for more information

sIg, surface immunoglobulin; CD, cluster of differentiation; FMC, epitope on CD20.

† , Score > 3: strong correlation with a diagnosis of CLL; score = 3: atypical CLL; score < 3 unlikely for chronic lymphocytic leukaemia and more likely for other B-cell lymphoproliferative disorders.

### Data analysis

To assess whether the MFI difference observed at 24 h, 48 h, 72 h or 96 h during storage of the blood samples at RT or 4 °C were different from their initial baseline values (at < 12 h, RT), a two-factor analysis of variance was used. When the two-factor analysis of variance test showed significant changes, a post-hoc two-tailed T-test was used to determine which pairwise comparison of means (storage time versus storage temperature) contributed to the overall significant difference. Analysis was performed using Microsoft® Excel® (Microsoft Corporation, Redmond, Washington, United States).

## Results

### Samples included

Twelve samples were initially screened, although only 10 samples were included in the final analysis. Two samples were excluded because of ‘atypical’ CLL scores calculated at baseline.

### Median fluorescence intensity and median fluorescence intensity difference

Irrespective of storage temperature, some markers, including CD23, CD200 and sIgM, showed > 10% decrease in MFI difference (CD23: *p* = 0.003; CD200: *p* = 0.005; and sIgM: *p* = 0.07) already at 24 h. The markers that did not show a significant change in MFI difference in relation to time or storage temperature were CD20, CD79b, sKappa and sLambda.

At RT, the MFI difference for CD5 showed a statistically significant decrease only at 96 h (*p* = 0.028), whereas the samples kept at 4 °C showed no significant change in MFI difference over time (*p* = 0.08 at 96 h).

CD23 showed statistically significant changes with storage time (*p* ≤ 0.001) and storage temperature (*p* = 0.025), with a decrease in the MFI difference of > 15% from baseline already at 24 h for both RT (*p* = 0.015) and 4 °C (*p* = 0.032).

**FIGURE 3:**
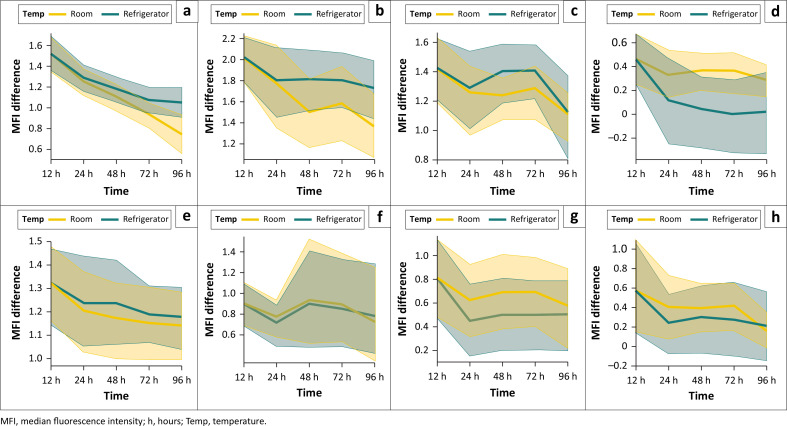
Changes in median fluorescence intensity difference over time for each marker, CD23 (a), CD200 (b), CD5 (c), SlgM (d), CD20 (e), CD79b (f), Kappa (g) and Lambda (h), at the different storage temperatures at Universitas Academic Hospital, Bloemfontein, South Africa, January 2022 to March 2023. The solid line represents the means of the median fluorescence intensity difference and the shaded area indicates the range of results at the various time points.

CD200 was more sensitive to time (*p* = 0.05) compared to temperature (*p* = 0.07), with > 25% decrease in MFI difference at 48 h (*p* = 0.013) in the RT samples. However, the samples kept at 4 °C showed no significant change in MFI difference over time (*p* = 0.08 at 96 h). Storage temperature rather than time played a statistically significant role in sIgM expression (*p* = 0.006). It was noteworthy that the sIgM MFI difference was more stable at RT, while a decrease of > 90% was observed in the 4 °C samples at 48 h (*p* = 0.022). In addition, sKappa and sLambda were more stable at RT, compared to samples kept at 4 °C. There was, however, no clinically significant decrease in the 4 °C samples, even at 96 h (*p* = 0.11 for sKappa and *p* = 0.13 for sLambda) (Figure 3).

### Chronic lymphocytic leukaemia scores

**FIGURE 4:**
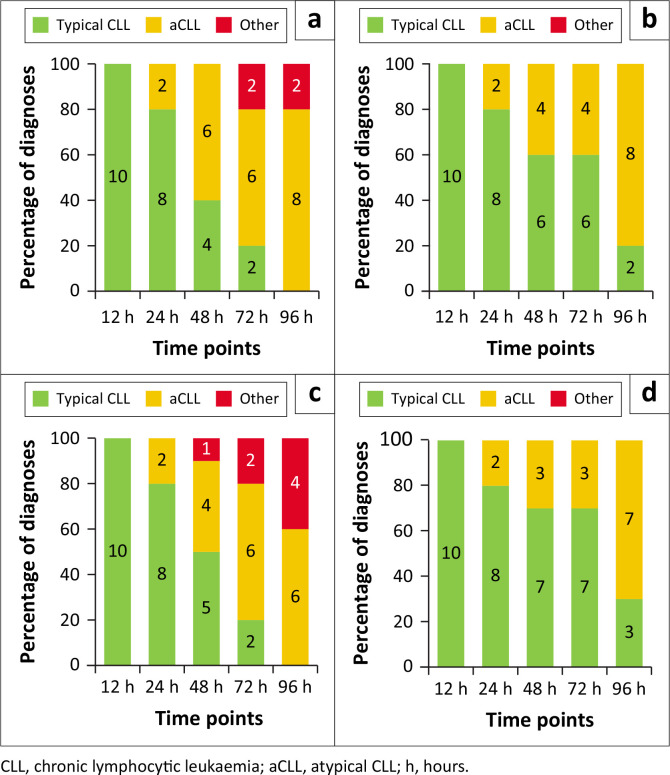
Temporal evolution of the chronic lymphocytic leukaemia diagnoses (from baseline to 96 h) at Universitas Academic Hospital, Bloemfontein, South Africa, January 2022 to March 2023. **Top:** Moreau score at room temperature (a) and 4 °C (b). **Bottom:** Adapted Matutes score at room temperature (c) and 4 °C (d).

To determine whether storage time or temperature would affect the CLL diagnostic scores, the Moreau and adapted Matutes scores were calculated at various time points. A two-way analysis of variance was performed to compare the effect of storage time and temperature on the MFI for the markers included in the CLL scores. Both the Moreau and the adapted Matutes scores showed statistically significant variation with temperature (Moreau: *p* = 0.007; and adapted Matutes: *p* = 0.005) and time (Moreau: *p* ≤ 0.001; and adapted Matutes: *p* ≤ 0.001). A post-hoc analysis confirmed 48 h to be the most significant time point for both scoring systems in RT samples (Moreau: *p* = 0.002; and adapted Matutes: *p* = 0.011) as well as the 4 °C samples (Moreau: *p* = 0.018; and adapted Matutes: *p* = 0.040). The Moreau score of most RT samples (*n* = 6) and four of the 4 °C samples changed to atypical CLL at 48 h. A majority shift (*n* = 8 for the Moreau score; *n* = 7 for the adapted Matutes score) in diagnosis from typical CLL to atypical CLL for the 4 °C samples was only seen at 96 h (Figure 4).

## Discussion

In this study, we observed statistically significant changes in CD5, CD23, and CD200 antigen expression, with antigen stability notably improved under refrigerated conditions. The majority of samples stored at RT (*n* = 6) and only four of the refrigerator samples displayed a shift in diagnosis towards aCLL at the 48 h mark. However, a majority shift in diagnosis from CLL to aCLL/non-CLL for the refrigerator samples was only evident after a 96 h storage duration.

The International Council for Standardisation of Haematology and the International Clinical Cytometry Society emphasise the need for standardised pre-analytical procedures, with recommendations that samples be analysed within 48 h when stored at RT.^20^ However, Bain et al. suggest that samples should ideally be analysed within 24 h if transported and stored at RT, particularly when referral to central laboratories is involved.^27^ Other studies, like those by Diks et al.,^19^ have highlighted the impact of pre-analytical handling on data quality, noting that sample processing should be tailored to the cell type and clinical context. Notably, most of these investigations used samples from healthy donors, not individuals with haematological malignancies.^17,18,19^ Comparative studies on optimal pre-analytical conditions for B-LPD specimens remain limited,^19^ underscoring a gap that our research set out to address.

Our findings align with the EuroFlow consortium, which examined pre-analytical variables on samples from patients with haematological malignancies and suggested that protocols may need adjustments based on sample type and markers analysed.^28^ Notably, we found that CLL antigen expression is better preserved at refrigerator temperatures than RT, which contrasts with previous reports.^27^ Among the assessed markers, CD23 and CD200 showed statistically significant expression changes over time, with better stability at refrigerated temperatures. Different definitions of RT worldwide could partially explain these discrepancies. For instance, RT standards range from 15 °C to 30 °C globally, depending on climate and regulatory guidelines.^29,30^ In South Africa, where this study was conducted, RT typically falls between 21 °C and 26 °C,^31^ with 22 °C ± 2 °C advised for blood-handling facilities.^31,32^ Additionally, structural similarities between CD23 and CD200 – both containing immunoglobulin-like domains – might influence their stability under varying conditions.^33,34^

Our study suggests that maintaining samples at refrigerator temperature, rather than RT, may better preserve sample integrity and accuracy in flow cytometric analyses. This principle applies not only to flow cytometry samples but has also been shown to be true for full blood count parameters when processing is delayed.^35^ Interestingly, sIgM levels decreased significantly under refrigerated conditions, potentially because of its low baseline expression in CLL cells, making it more susceptible to degradation. Alternatively, Guo et al. found that interleukin-4 (not assessed in this study) could increase sIgM expression in CLL,^36^ while Jackson et al. reported lower interleukin-4 levels at 4 °C compared to RT,^37^ potentially explaining the observations.

Although the evaluation of CD200 is not a mandatory criterion in CLL diagnosis as defined by the World Health Organization,^3^ its inclusion has been advocated in the EuroFlow guidelines, and previous research has attested to its high diagnostic utility, especially in distinguishing CLL from mantle cell lymphoma.^38^ The adapted Matutes score represents a four-marker scoring system, which incorporates CD200, streamlining the antibody requirements for CLL diagnosis, and offering valuable advantages in resource-constrained settings.^10^ In the current research, the adapted Matutes score exhibited greater diagnostic accuracy for CLL than the Moreau score when dealing with samples subjected to delayed analysis. Arguably, the inclusion of fewer markers susceptible to time and temperature-dependent changes may translate to a more robust scoring system with heightened accuracy.

The influence of storage conditions on the percentage antigen expression and MFI of markers employed in the CLL scoring systems allowed a shift in the calculated scores to support a diagnosis of aCLL or even another B-LPD. Notable deviations in the adapted Matutes and Moreau scores were discerned in two samples after only 24 h of storage, irrespective of the temperature setting. This may partly be explained by the elevated white cell counts (± 200 × 10^9^/L) observed in these two samples. However, underlying genetic aberrations may also have contributed.

The clinical significance of aCLL remains debatable, and in the absence of a standardised and universally accepted definition for aCLL,^14^ the term is often used more broadly to describe cases with an unconventional immunophenotype and is unlikely to represent a single biological entity.^15^ As such, the term ‘B-LPD other than CLL’ may be more appropriate to prevent the inadvertent exclusion of other B-LPD subtypes. With treatment modalities revolutionising to more targeted therapies, the distinction between CLL and other B-LPDs invokes significant treatment implications.

### Limitations

Budgetary constraints imposed limitations on the number of samples subjected to analysis. Despite only including 10 patients in our study, each patient’s sample underwent flow cytometric analysis nine times, encompassing baseline assessments and subsequent evaluations at four different time points, both under refrigerator and RT conditions.

We enrolled patients exhibiting morphologically detectable CLL and a typical baseline immunophenotype, irrespective of their treatment status. It is important to note that the impact of treatment could potentially alter the expression of specific markers in CLL.

For our analysis, we adhered to specific fluorochromes according to the EuroFlow guidelines.^7,22^ These findings may not be universally applicable to products from alternative manufacturers.

Samples were collected in EDTA tubes. Various studies have indicated that the stability of cell surface marker expression and population distribution is greater in sodium-heparin blood compared to EDTA blood.^19,20^ However, it should be noted that storing sodium-heparin samples is associated with a more rapid decline in leukocyte counts over time. The use of whole blood fixation strategies (Cyto-Chex®, TransFix®) to preserve cell populations in stored samples intended for flow cytometric analysis was beyond the scope of this study. Yet, these reagents have been shown to reduce signal intensity.^19,20^

Future studies could involve larger cohorts, encompassing diverse haematological malignancies. In addition, correlations between disease progression, molecular abnormalities, or treatment interventions and alterations in antigen expression of cells could be investigated.

### Conclusion

In conclusion, this study showed that storage time and storage temperature impact the stability of surface markers required for the diagnosis of CLL, which we can translate to our clinical setting. To ensure reliable results, referral samples expected to reach the laboratory more than 48 h after collection must be maintained at refrigerator temperatures during storage and transportation. While our study specifically focused on CLL as a proof of concept, the implications of our findings likely extend beyond the markers employed in CLL diagnosis and the pre-analytical phase may need to be customised to the clinical application.
